# Kinetic control of nascent protein biogenesis by peptide deformylase

**DOI:** 10.1038/s41598-021-03969-3

**Published:** 2021-12-27

**Authors:** Lena A. K. Bögeholz, Evan Mercier, Wolfgang Wintermeyer, Marina V. Rodnina

**Affiliations:** Department of Physical Biochemistry, Max Planck Institute for Biophysical Chemistry, 37077 Göttingen, Germany

**Keywords:** Enzyme mechanisms, Biochemistry, Molecular biology, Post-translational modifications, Translation

## Abstract

Synthesis of bacterial proteins on the ribosome starts with a formylated methionine. Removal of the N-terminal formyl group is essential and is carried out by peptide deformylase (PDF). Deformylation occurs co-translationally, shortly after the nascent-chain emerges from the ribosomal exit tunnel, and is necessary to allow for further N-terminal processing. Here we describe the kinetic mechanism of deformylation by PDF of ribosome-bound nascent-chains and show that PDF binding to and dissociation from ribosomes is rapid, allowing for efficient scanning of formylated substrates in the cell. The rate-limiting step in the PDF mechanism is a conformational rearrangement of the nascent-chain that takes place after cleavage of the formyl group. Under conditions of ongoing translation, the nascent-chain is deformylated rapidly as soon as it becomes accessible to PDF. Following deformylation, the enzyme is slow in releasing the deformylated nascent-chain, thereby delaying further processing and potentially acting as an early chaperone that protects short nascent chains before they reach a length sufficient to recruit other protein biogenesis factors.

## Introduction

During biosynthesis on the ribosome, proteins undergo a variety of modifications before they reach their mature form. In bacteria, the N-terminal methionine initially carries a formyl group, which, for most proteins, is removed by peptide deformylase (PDF)^1^ co-translationally when the nascent chain emerges from the peptide exit tunnel of the ribosome. Exceptions are secretory proteins and membrane proteins that recruit the signal recognition particle (SRP), which prevents this deformylation^2^. PDF catalyzes the hydrolytic cleavage of the formyl group from the N-terminal formylmethionine (fMet) of the nascent peptide, yielding formic acid as reaction product. The enzyme is a small α + β protein with two lobes folding around the active site^3^ containing a metal cofactor, Fe(II)^4^, which is coordinated by cysteine and two histidine residues^5^. For in vitro studies, Fe(II) is commonly replaced with a less oxidation-sensitive metal ion such as Co(II), which does not alter the enzyme’s structure or activity^6,7^. Deformylation is essential for bacterial viability^8^ and is required for subsequent N-terminal methionine cleavage by methionine aminopeptidase (MAP), an event which occurs for about half of all proteins^9,10^. As human mitochondrial PDF is upregulated in cancer cells, PDF is a potential drug target and PDF inhibitors such as actinonin are being explored for potential use in anticancer treatment^11–13^.

The actions of different protein biogenesis factors are coordinated by the ribosome, which provides a binding platform for PDF, MAP, and SRP in the vicinity of the tunnel exit where the N-terminus of a nascent protein emerges. SRP rapidly scans ribosomes until it encounters a nascent protein displaying an SRP-specific signal sequence, to which SRP binds tightly^14^. PDF interacts via its C-terminal helix with ribosomal protein L22 near the tunnel exit^15–17^, and its binding site overlaps with that of MAP, resulting in competitive binding^16,18^. Other factors, including the chaperone trigger factor (TF) or SRP, can bind to the ribosome at the same time as PDF in a non-competitive manner^16,17,19^; simultaneous binding of PDF, TF and MAP may impair the function of TF^17^.

During ongoing translation in the cell, the N-terminus of the nascent polypeptide has to emerge from the exit tunnel of the ribosome prior to deformylation. With stalled RNCs, maximal PDF activity is achieved when the chain length is about 70 amino acids^2,20^, although some activity can be detected with chains as short as 50 amino acids^10,21,22^. Recent kinetic measurements of MAP activity, which depend on the preceding action of PDF, suggest that the rate of nascent peptide deformylation is in the range of 1 s^−1^, which is slow compared to the rate of protein synthesis (in the range of 10 s^−1^) and limiting for the subsequent MAP reaction^20^. The low deformylation rate on the ribosome is particularly surprising, given that the nascent peptide directly faces PDF upon emergence from the exit tunnel, which should contribute to a favorable pre-orientation effect^15^. Furthermore, kinetic studies of PDF-catalyzed deformylation using formylated peptides that mimic the N-terminus of nascent proteins, such as the chromogenic model substrate formyl-methionyl-leucyl-*p*-nitroaniline (fMLpNA)^7,23^, revealed deformylation rates of 20–40 s^−1^ and up to 1000 s^−1^ depending on the amino acid composition^7,23–27^. As direct measurements of PDF-catalyzed nascent-chain deformylation on the ribosome have not yet been reported, the kinetic mechanism of deformylation on the ribosome is not known, and the reasons underlying the slow reaction remain elusive.

In this paper, we dissect the kinetic mechanism of PDF action on the ribosome and, using ribosome-nascent-chain complexes (RNC) as substrates, we show that peptide deformylation is intrinsically rapid. However, a subsequent slow rearrangement step limits the rate of the PDF turnover. Thus, PDF may delay the recruitment of downstream ribosome-associated protein biogenesis factors to the nascent-chain and potentially hinder folding of the N-terminus to avoid misfolding events.

## Results

### Multiple-turnover RNC deformylation

**Figure 1:**
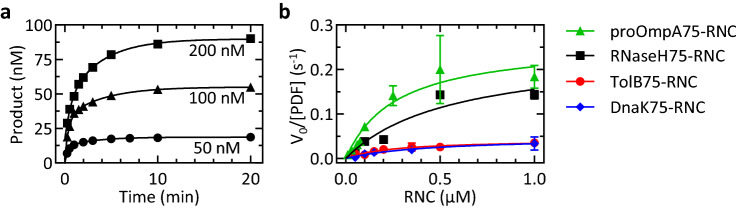
RNC deformylation at multiple turnover conditions. (**a**) Deformylation time courses measured at limiting PDF concentration (10 nM) and increasing concentration of RNaseH75-RNC, as indicated. (**b**) Michaelis–Menten plot. Initial velocities (*V*_*o*_) are normalized by the concentration of PDF (10 nM for RNaseH75-RNC, TolB75-RNC, DnaK75-RNC; 1 nM for proOmpA75-RNC). Michaelis–Menten fits (lines) yield the parameters summarized in Table 1. Error margins indicate standard errors determined from linear fitting of individual time courses.

We first determined the rate of nascent-chain deformylation at conditions of multiple turnover for PDF. We used RNCs carrying 75 amino acid-long nascent-chains, which is an optimal length for deformylation on the ribosome^2,20^ and a ^35^S-labeled fMet on the N-terminus on the nascent-chain. After incubation of RNCs with PDF and subsequent proteinase K (PK) digestion, f[^35^S]Met and [^35^S]Met were separated by thin-layer chromatography (TLC) and visualized by phosphoimaging (“Materials and methods”; Fig. S1). We measured time courses of deformylation (Fig. 1a) and determined Michaelis–Menten constants k_cat_ and K_M_ from the concentration-dependent initial velocities of four RNC substrates carrying different nascent peptides, proOmpA, RNaseH, TolB and DnaK (Fig. 1b; Table 1). Previous studies indicated that these substrates are efficiently deformylated albeit at different rates, making them suitable substrates to study^2^. We also examined the Michaelis–Menten kinetics of PDF acting on the dipeptide substrate fMLpNA for comparison (Table 1). For different RNCs, the K_M_ values are similar, within a factor of two, whereas k_cat_ values vary about sixfold, between 0.04 and 0.26 s^−1^. For all RNCs, k_cat_ values are at least 60 times lower than for model substrates (Table 1 and refs.^7,23,26^). The variations in k_cat_ observed for different RNCs can be explained by the effect of the N-terminal amino-acid sequence or structure on deformylation velocity^2,25^. The K_M_ values for the RNCs are about 100-fold lower than those for fMLpNA (Table 1), which in the simplest model, reflects stabilization of the PDF-nascent-chain complex by binding of PDF to the ribosome. The k_cat_/K_M_ values are in the same order of magnitude for RNCs and fMLpNA, and similar to the value obtained with a different RNC substrate^20^, indicating a similar catalytic efficiency on and off the ribosome, despite largely different k_cat_ and K_M_ values. The smaller k_cat_ values for RNCs compared to fMLpNA points towards a rate-limiting step in the mechanism of deformylation that is specific to RNCs.

**Table 1:** Michaelis–Menten parameters of deformylation by PDF.

Substrate	K_M_ (µM)	k_cat_ (s^−1^)	k_cat_/K_M_ (µM^−1^ s^−1^)
RNaseH75-RNC	0.6 ± 0.5	0.24 ± 0.09	0.4
proOmpA75-RNC	0.3 ± 0.1	0.26 ± 0.04	0.9
TolB75-RNC	0.25 ± 0.08	0.04 ± 0.01	0.2
DnaK75-RNC	0.5 ± 0.2	0.05 ± 0.01	0.1
fML-pNA	24 ± 6	16 ± 2	0.7

Errors represent standard errors of the fit using the Michaelis–Menten equation (Fig. 1b).

The deformylation of the formylated peptide fML-pNA was monitored photometrically (“Materials and methods”).

### Rapid single-round deformylation

**Figure 2:**
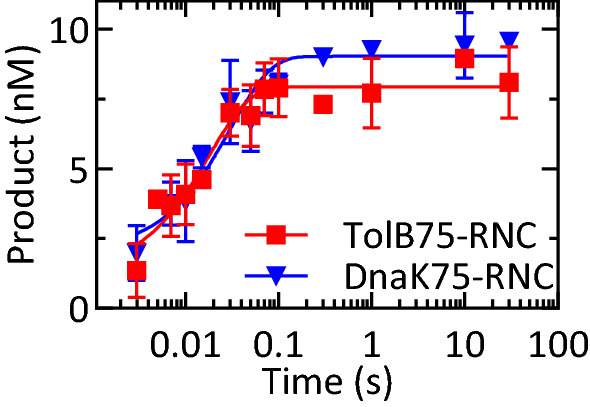
Pre-steady-state kinetics of RNC deformylation. DnaK75-RNC or TolB75-RNC (10 nM final concentration) were rapidly mixed with PDF at saturating concentration (25 µM) in a quench-flow apparatus at 37 °C. At the indicated times, samples were quenched, treated with PK, and analyzed by TLC (“Materials and methods”). Time courses were evaluated by fitting to a single-exponential function, yielding an apparent rate constant k_app_ (37 °C) = (48 ± 9) s^−1^. Error margins represent the standard deviation of three independent experiments.

To help identify the slow step, we measured the rate of deformylation at conditions where the reaction is not limited by enzyme turnover. To ensure that the binding of PDF to the ribosome is rapid compared to the chemistry step and that the substrate is converted to product in a single round, a large excess of PDF over RNC was used (Fig. 2). The reaction started upon rapid mixing of RNC and PDF in a quench-flow apparatus and, after the indicated incubation times, samples were quenched and analyzed by TLC to determine the product:substrate ratio (“Materials and methods”). Reaction end-level values quantified in parallel experiments indicated that 90 ± 2% of DnaK75-RNC and 78 ± 4% of TolB75-RNC was converted to product (Fig. S2). Time courses were fitted with single-exponential functions to obtain deformylation rates of 33 ± 8 s^−1^ for DnaK75-RNC and 60 ± 20 s^−1^ for TolB75-RNC (Fig. 2) which are not significantly different and were therefore averaged (k_app_ = 50 ± 20 s^−1^). These rates are comparable to the k_cat_ measured with the model dipeptide off the ribosome^7,23,26^ and 1000-fold higher than the k_cat_ values of the reaction on the ribosome measured at multiple-turnover conditions (Table 1). Thus, the chemistry step of the PDF-catalyzed deformylation is intrinsically rapid, and the rate-limiting step of the reaction on the ribosome must take place after hydrolysis.

### PDF dissociates rapidly from deformylated RNCs

**Figure 3:**
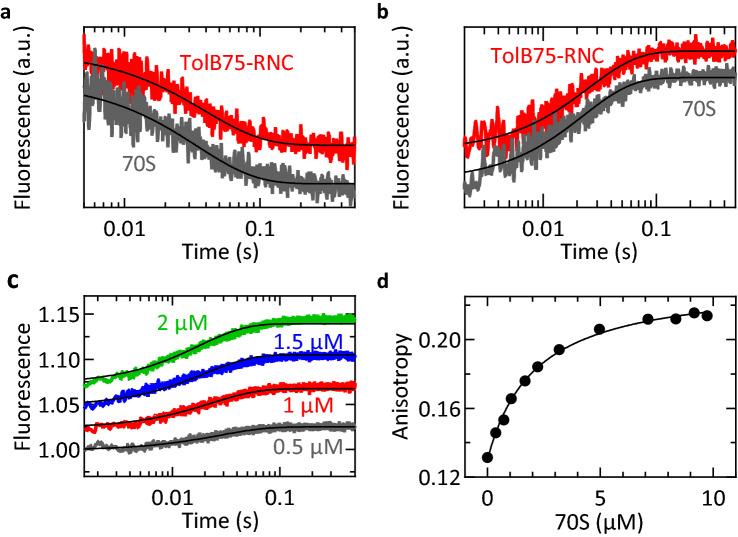
Interaction of PDF with TolB75-RNC and vacant 70S ribosomes. In (**a**,** b**) time courses are shifted on the Y-axis for visual clarity. (**a**) Time courses of PDF dissociation from the ribosome. Time courses of 70S–PDF(Bpy) or RNC–PDF(Bpy) (150 nM) dissociation upon addition of excess unlabeled PDF (15 µM) were measured in a stopped-flow apparatus at 10 °C. Eight technical replicates were averaged for each trace and smooth lines represent single-exponential fits. (**b**) Association time courses. Binding was monitored upon rapid mixing of PDF(Bpy) (300 nM) with 70S ribosomes or TolB75-RNC (420 nM) at 10 °C. Six technical replicates were averaged and evaluated by single-exponential fitting (smooth lines). (**c**) Binding of PDF(Bpy) (100 nM) to 70S ribosomes (0.5–2 µM) monitored by fluorescence intensity changes at 10 °C. Each time course represents an average of seven technical replicates. The smooth lines result from global fitting to a one-step binding model (Table 2). (**d**) Binding of PDF to 70S ribosomes monitored by changes in fluorescence anisotropy of PDF(Bpy) (200 nM). The data were fitted to a hyperbolic function.

For many enzymes, product dissociation is rate-limiting for the turnover reaction. To test whether this could explain the slow turnover of PDF, we incubated TolB75–RNC with PDF to deformylate the nascent-chain and then measured dissociation of the PDF–RNC complex via fluorescence of Bodipy (Bpy) attached to the C-terminus of PDF (“Materials and methods”). Upon mixing the labeled PDF(Bpy)–RNC complex with an excess of unlabeled PDF in the stopped-flow apparatus, the fluorescence intensity decreases rapidly, indicating fast dissociation of PDF(Bpy) from the complex. The rate constant of dissociation obtained by single-exponential fitting of this trace is 27 ± 3 s^−1^ at 10 °C (Fig. 3a). Following previous studies^18^ we used lower reaction temperatures for these dissociation experiments because no signal change was observed at 37 °C. Assuming the typical temperature dependence of enzymatic reactions, the dissociation is expected to be faster at 37 °C. Since dissociation of PDF from deformylated RNCs is rapid, it cannot explain the slow PDF turnover observed under multiple-turnover conditions. PDF dissociates from vacant ribosomes at a rate of 33 ± 3 s^−1^, which is not significantly different from the rate observed with deformylated RNCs (Fig. 3a). This suggests that here we monitor a step in which PDF dissociates from its contact site on the ribosome, rather than the release of PDF from the nascent-chain.

### PDF binding to RNCs is rapid and independent of the nascent peptide

To identify the rate-limiting step, we set out to elucidate the kinetic mechanism of RNC deformylation. We first monitored PDF binding to formylated TolB75-RNC using fluorescence-based stopped-flow experiments (Fig. 3b). Binding was rapid (40 ± 3 s^−1^) and indistinguishable from binding to vacant 70S ribosomes (44 ± 2 s^−1^) (Fig. 3b) at 10 °C, again indicating that association of PDF with the RNC is dominated by interactions with the ribosome, rather than with the nascent-chain. This allowed us to use vacant 70S ribosomes to determine rate constants for PDF binding and dissociation, which are likely representative for PDF recruitment to the RNCs as well (RNC titrations in a comparable concentration range are not feasible). The use of 70S ribosomes also simplifies the kinetic mechanism, since there is no nascent-chain present to be deformylated, and the system reaches equilibrium after PDF binding.

To determine rate constants of ribosome-PDF complex formation, PDF(Bpy) was rapidly mixed with 70S ribosomes at increasing concentrations in the stopped-flow apparatus at 10 °C. The Bpy fluorescence changes accompanying complex formation (Fig. 3c) were globally fitted using the KinTek Explorer software^28,29^ according to a one-step binding model. This fitting yielded a binding rate constant k_1_ of 14 ± 2 µM^−1^ s^−1^ and a reverse rate constant k_−1_ of 29 ± 2 s^−1^; the latter is the same as determined in chase experiments (Fig. 3a). The K_d_ for this interaction was determined independently by equilibrium titration while monitoring changes in fluorescence anisotropy of PDF(Bpy) upon addition of 70S ribosomes (Fig. 3d). The measured K_d_ of 2.1 ± 0.2 µM is in agreement with the value determined from kinetic constants (K_d_ = k_−1_/k_1_ = 2.1 ± 0.4 µM) and with values reported in previous studies: 1.5 µM^19^, 1.8 ± 0.9 µM (12 °C)^15^ or 2.6 µM (10 °C)^18^. To facilitate comparison with deformylation rates, rate constants k_1_ and k_−1_ measured at 10 °C were extrapolated to 37 °C using a previously established method^30^, which allows to calculate rate constants with the correction factor obtained from temperature dependence of a key step on the pathway (“Materials and methods”). As a reference reaction, we used single-round deformylation with a rate of 24 ± 3 s^−1^ at 10 °C (Fig. S2) compared to 60 ± 20 s^−1^ at 37 °C (Fig. 2). From these values, the extrapolated rate constants were 40 ± 20 µM^−1^ s^−1^ for binding and 70 ± 30 s^−1^ for dissociation (Table 2), which are 100-fold larger than the reported k_cat_ values and, therefore, not rate-limiting.

**Table 2:** Kinetic parameters of PDF binding to 70S ribosomes.

	10 °C	37 °C
*k*_1_ (µM^−1^ s^−1^)	14 ± 2	40 ± 20
*k*_−1_ (s^−1^)	29 ± 2	70 ± 30
K_d,calc_ (µM)	2.1 ± 0.4	2 ± 1
K_d,anisotropy_ (µM)	2.1 ± 0.2	n.d.

Rates measured at 10 °C were extrapolated to 37 °C and K_d,calc_ values were calculated from rate constants (“Materials and methods”).

K_d,anisotropy_ was measured by 70S ribosomes titration at 10 °C (Fig. 3d). n.d., not determined.

### Reversibility of deformylation

**Figure 4:**
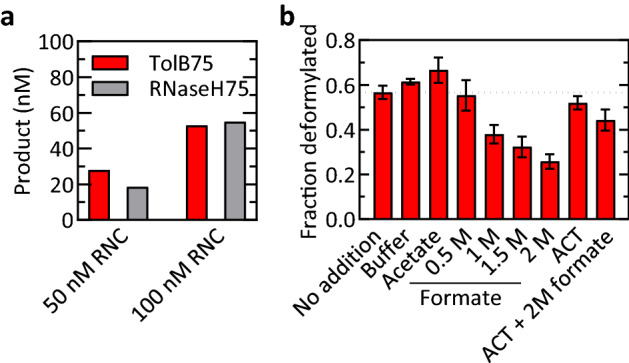
Reversal of deformylation. (**a**) End level of deformylation at different RNC concentrations. TolB75-RNC and RNaseH75-RNC were incubated with PDF (10 nM) for 20 min at 37 °C. (**b**) Reversibility. After incubation of TolB75-RNC (50 nM) with PDF (1 µM) for 10 min (‘No addition’), additions were made as indicated. The PDF inhibitor actinonin (ACT; 2 mM) was included where indicated. Deformylation levels measured after 30 min further incubation are depicted. Error margins represent the standard deviation of three independent experiments.

We noted that in all of our deformylation experiments, the end levels were consistently below 50% in multiple-turnover experiments, regardless of the RNC or concentration (Figs. 1a, 4a) and in agreement with previous studies^2,20^. The 50% end level was not due to loss of enzyme activity, because addition of new RNC to a multiple-turnover deformylation reaction that had already reached a plateau resulted in the appearance of more deformylated product (Fig. S3a). Also, the RNCs are more than 50% functional, as indicated by the end levels of reactions at high PDF concentrations, which reached 80–90% for different RNCs (Fig. S3b). One possibility to explain the 50% reaction endpoint is that hydrolysis is reversible, and thus the end level is defined by the equilibrium between forward deformylation and backward reformylation. Consistent with this idea, higher end levels were observed at higher PDF concentrations (Fig. S3b), since addition of enzyme would tend to shift the equilibrium in the direction of deformylation. To test this hypothesis directly, we added product to a completed deformylation reaction, which would drive a reversible reaction in the opposite direction, causing reformylation. We achieved this by addition of formate to generate small amounts of formic acid, a product of deformylation, while maintaining a constant pH, in accordance with the Henderson-Hasselbalch equation. This allowed us to implement a standard approach for investigation of product inhibition in steady-state enzyme kinetics. While high formic acid concentrations are unlikely to emerge in vivo, this approach allowed us to calculate the internal equilibrium of the reversible deformylation step. This internal equilibrium is an inherent property of the enzyme, reducing the net k_cat_ value by extending the delay prior to PDF turnover. The test was carried out under multiple-turnover conditions, where deformylation of TolB75-RNC was performed until the end level was reached (Fig. 4b, “No addition”) and then formate, the deformylation product, was added. This caused the expected reduction of deformylated product, indicating that deformylated RNC was partly reformylated, as expected for a reversible reaction. In the presence of actinonin, a competitive PDF inhibitor (Fig. S4), the effect of formate is reduced substantially, indicating that reformylation is catalyzed by PDF. For comparison, the addition of buffer or acetate did not lead to a decrease of the deformylated product. Thus, peptide deformylation by PDF on the ribosome is reversible.

### The deformylated nascent chain rearranges slowly

**Figure 5:**
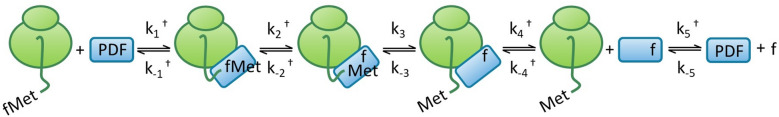
Minimal kinetic model of deformylation. Rate constants obtained from association experiments (Table 2), estimated from single-turnover experiments (Fig. 2) or found to be rapid but non-defined were kept fixed (^†^) during fitting. The remaining time courses were globally fitted to multiple- and single-turnover kinetics from TolB75-RNC, DnaK75-RNC, RNaseH75-RNC and proOmpA75-RNC (Fig. 6). f represents the formyl group.

Next, we used the combined data to describe a minimal kinetic mechanism of RNC deformylation by PDF and explain the slow multiple-turnover behavior (Fig. 5). In the simplest model, PDF binding to and dissociation from the ribosome can be described as a rapid one-step process (Fig. 3). The following chemistry step is reversible (Fig. 4) and much faster than the slow turnover of PDF measured at multiple-turnover conditions (Fig. 2), indicating the existence of an additional slow step after deformylation but prior to dissociation of PDF, which is also rapid (Fig. 3a). Finally, reversible formate dissociation from PDF is considered as a separate reaction, as proposed by mechanistic studies on model peptides^31^. We note that this minimal model does not take into account a potential step at which the nascent peptide is recruited to PDF before deformylation. This step must be fast, because the following deformylation step is rapid and not rate-limiting, but it may contribute to the stabilization of PDF on RNC, which is not captured in our model.

Next, we estimated the rate constants of individual steps based on the available kinetic information (Table 2). To describe the kinetics of enzyme binding and dissociation, we used the values for rate constants k_1_ and k_−1_ determined from binding and dissociation experiments carried out at 10 °C (Fig. 3), and extrapolated them to 37 °C (Table 2). Since PDF binding and dissociation were indistinguishable for ribosomes and RNCs, we apply the same values of k_1_ and k_−1_ for all RNCs and, furthermore, we infer the same rate constants for PDF interaction with ribosomes after deformylation (i.e. k_−4_ = k_1_, k_4_ = k_−1_). The forward and reverse rate constants of deformylation, k_2_ and k_−2_, were determined from the apparent rates and end levels of deformylation at single turnover conditions, where one can assume that the following reactions do not contribute significantly (“Materials and methods”).

To estimate deformylation rate constants for the four different RNCs, we used the average apparent rate of deformylation of about 50 s^−1^ for all constructs, and took into account the slightly different end levels for each RNC. For TolB75 and DnaK75, these values were taken from Fig. 2, while end levels for RNaseH75-RNC (80%) and proOmpA75-RNC (88%) were determined in PDF titration experiments (Fig. S3b). The resulting forward rate constants k_2_ were statistically indistinguishable for the four substrates, on average 40 s^−1^ and calculation of the reverse rate constants k_−2_ yielded values of 5–11 s^−1^ depending on the RNC (Table 3). These values provide reasonable estimates for deformylation rate constants based on the single-turnover reaction alone and with the implicit assumption that the following step is slow compared to single-round deformylation. This assumption is clearly supported by comparison of the single- and multiple-round turnover rates. Values of k_2_ and k_−2_ were therefore fixed in the subsequent global fitting. In order to test the robustness of the fit, we also fitted the k_−2_ values together with all other free parameters and found that the values did not change, but rather increased the uncertainty of the fit due to interdependence between rate constants.

**Table 3:** Overview of globally fitted rate constants.

	TolB75	DnaK75	RNaseH75	proOmpA75
k_1_ (µM^−1^ s^−1^)^a^	40 ± 20			
k_−1_ (s^−1^)^a^	70 ± 30			
k_2_ (s^−1^)^a^	40 ± 5			
k_−2_ (s^−1^)^a^	11 ± 4	5 ± 2	10 ± 3	6 ± 2
k_3_ (s^−1^)	0.15 ± 0.04	0.09 ± 0.02	0.5 ± 0.06	n.d.
k_−3_ (s^−1^)	50 ± 20	22 ± 8	40 ± 10	n.d.
k_4_ (s^−1^)^a^	70 ± 30			
k_−4_ (µM^−1^ s^−1^)^a^	40 ± 20			
k_5_ (s^−1^)^a^	200			
k_−5_ (µM^−1^ s^−1^)^b^	200 ± 20			

Errors represent standard errors from global fit analysis or error margins determined from experiments (^a^).

^a^Value fixed during fitting.

^b^Value shared between all RNCs. n.d., not defined.

**Figure 6:**
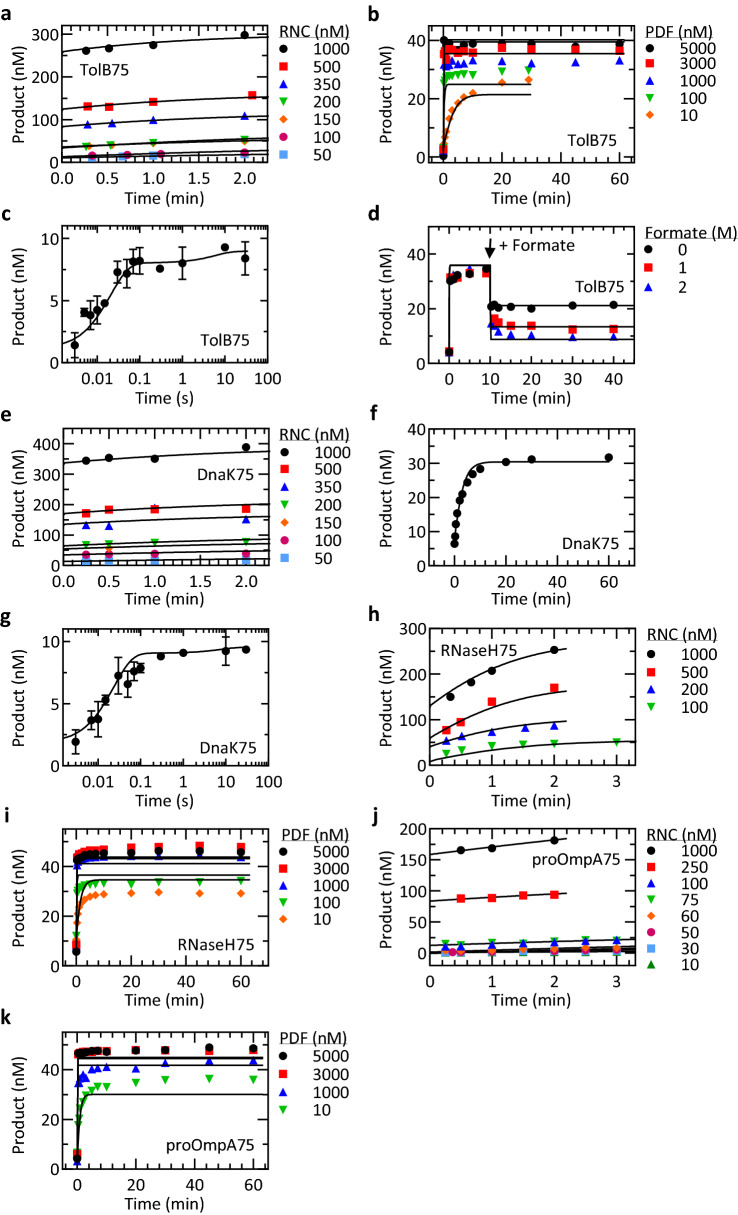
Global fit. All data of TolB75-RNC, DnaK75-RNC, RNaseH75-RNC and proOmpA75-RNC were used for global fitting in KinTek Explorer (“Materials and methods”). TolB75-RNC: (**a**) Multiple-turnover kinetics; (**b**) PDF titration; (**c**) Single-turnover deformylation kinetics; (**d**) Deformylation with addition of formate. DnaK75-RNC: (**e**) Multiple-turnover kinetics; (**f**) Extended time course with PDF (10 nM) and RNC (50 nM); (**g**) Single-turnover kinetics. RNaseH75-RNC: (**h**) Multiple-turnover kinetics; (**i**) PDF titration. proOmpA75-RNC: (**j**) Multiple-turnover kinetics; (**k**) PDF titration. Solid lines depict the simulated concentrations based on the global fitting results of this figure. Error margins in (**c**,**g**) are standard deviations of independent experiments (n = 3 (TolB75) and n = 4 (DnaK75)).

The remaining rate constants (k_3_, k_−3_, k_5_, k_−5_) could not be determined analytically. To provide values for these rate constants and assess the feasibility of the proposed kinetic mechanism, we carried out global fitting using the model in Fig. 5, multiple-turnover and single-turnover experiments for all substrates, and formate-driven formylation of TolB75-RNC, all performed at 37 °C. Initial fits with all undetermined parameters free to change were poorly defined due to the completely reversible reaction and high interdependence of rate constants in the fit. In particular, global fitting indicated that formate dissociation from PDF must be fast, although the value could not be determined with precision. As this is clearly not a ribosome-dependent rate-limiting step, we fixed the respective rate constant to 200 s^−1^ in subsequent fitting. Reverse rates k_−5_ were then linked (i.e. considered to be identical) for different substrates, because formate binding to PDF is not expected to differ in the presence of different RNCs. With these constrains, we obtained good quality fits for all data sets (Fig. 6) and defined values for all rate constants, although the high interdependence between rate constants k_3_, k_−3_ and k_−5_ resulted in rather large error margins (Table 3).

The fitting provides estimates for the rate-limiting step of PDF turnover, which has a low forward rate (k_3_) and is readily reversible (k_−3_). This step is about fivefold faster for RNaseH75 than it is for TolB75 and DnaK75 (Table 3), which reflects the trend observed in k_cat_ values measured for these substrates (Table 1). The values for k_3_ are 2–5 times larger than the respective k_cat_ values due to the reversibility of the deformylation step, with the large values of k_−3_ decreasing the net rate in the direction of deformylated RNCs. Overall, the results of global fitting are consistent with the minimal kinetic model and the presence of a rate-limiting rearrangement after deformylation.

### The nascent-chain is deformylated upon emergence from the ribosome

**Figure 7:**
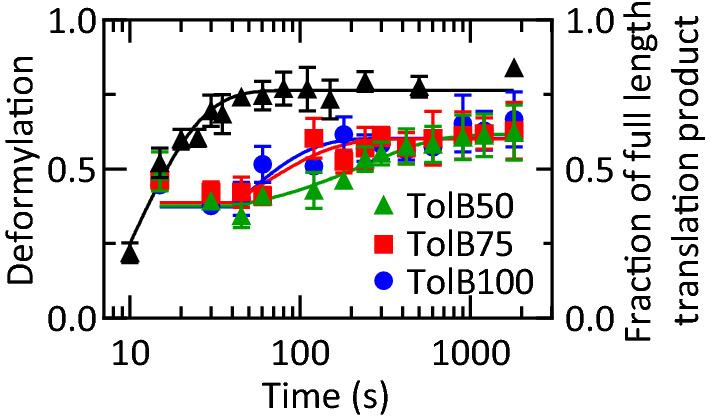
Co-translational deformylation of RNCs. Translation of TolB mRNA constructs of increasing length (50, 75, and 100 codons) was started with initiation complex (50 nM) by adding the components of the translation system and PDF (2 μM). Deformylation (colored symbols) is plotted on the left Y-axis, and translation of TolB50 mRNA (black triangles) on the right Y-axis. Co-translational deformylation time courses were fitted to delay-exponential equations (“Materials and methods”), and the rates of the multiple turnover phase were 0.004 s^−1^ (TolB50), 0.017 s^−1^ (TolB75), and 0.024 s^−1^ (TolB100). One-way ANOVA indicated higher rates for TolB75 and TolB100 compared to TolB50 (p = 0.018 and 0.0001, respectively), but no significant difference between ToB75 and TolB100 (p = 0.20). Error bars represent the standard deviation of three independent experiments.

Since deformylation in the cell occurs co-translationally, we also examined whether active translation influences deformylation kinetics. We translated TolB mRNA encoding 50, 75, or 100 N-terminal amino acids in the presence of PDF (Fig. S5) and followed nascent-chain deformylation concomitantly with protein synthesis after confirming that the addition of PDF does not alter translation kinetics (Fig. S6). At cellular PDF concentration (2 µM^24^), co-translational deformylation is extremely rapid on about 40% of RNCs (green triangles in Fig. 7), which are deformylated as soon as they reach the expected length of 50 aa (black triangles in Figs. 7, S5a). The end level of this rapid deformylation reaction is independent of the final nascent-chain length, suggesting that the action of PDF in this phase is only limited by the emergence of the nascent-chain from the exit tunnel. An additional 25% of RNCs are deformylated more slowly, and the rate of deformylation is somewhat lower for TolB50- than for TolB75- and TolB100-RNC, consistent with previous studies that indicated a length-dependent deformylation for purified RNCs at multiple-turnover conditions^2,20^. While rapidly deformylated RNCs result from single-round PDF action, it is likely that the slower phase of deformylation is determined by PDF turnover, as observed on stalled RNCs. By analogy with stalled RNC, the rate-limiting step of the multiple-turnover reaction is likely a rearrangement of the PDF-RNC complex after deformylation but before PDF release. This step would, therefore, also limit the rate of the nascent-chain handover from PDF to MAP on the fraction of RNCs which are deformylated rapidly.

## Discussion

The kinetic analysis of PDF activity on RNCs has revealed a reversible reaction with predominantly fast steps for binding and dissociation and a slow rearrangement, which takes place after deformylation and limits the rate of PDF dissociation from nascent peptides. Previous work has indicated that PDF has a similar affinity to ribosomes and RNCs carrying a short, non-exposed nascent-chain in the ribosomal exit tunnel^18^. Here we show that even when the nascent-chain is accessible for PDF, the initial binding kinetics is the same as with vacant ribosomes. Docking of the N-terminus into the active site of PDF is apparently rapid since it is not identified as an additional kinetic step, but it can be slowed or prevented by binding to SRP when the nascent-chain carries an SRP-specific signal anchor sequence^2,20^. The affinity of PDF for ribosomes and RNCs is in the range of 2 µM, so given cellular concentrations of ribosomes (20–45 μM depending on the growth rate^32^) and PDF (2 μM^24^), the majority of the PDF molecules are expected to be bound to ribosomes. However, rapid association and dissociation ensures that PDF can rapidly scan ribosomes and efficiently access RNCs that require deformylation. Although the area around the ribosomal tunnel exit also serves as a binding platform for other ribosome-associated protein biogenesis factors, PDF binding is not affected by either SRP or TF, indicating that scanning by PDF should proceed unabated in vivo in the presence of the other factors^33^. MAP, however, competes with PDF on the ribosome^16,18^, which will prevent PDF from rebinding to nascent-chains recruited to MAP and, therefore, enhance scanning of other ribosomes.

In the cell, where ribosomes are in excess over PDF, only a small fraction of ribosomes will be bound to PDF at a given time. Based on the rate constants determined here, PDF binds one ribosome every 0.3–2 ms on average, depending on the ribosome concentration, and the average residence time is 15 ms, allowing each PDF molecule to sample about 60 ribosomes in one second. At this rate, it takes 0.2 to 1 s for the roughly 1300 molecules of PDF to scan all 10,000–70,000 ribosomes in the cell. During this time, a nascent-chain can be elongated by up to 20 amino acids. Since nascent-chains suitable for deformylation are between 50 and 120 amino acids in length^2,20^, scanning is fast enough to reach all accessible formylmethionines within the available time window.

The reversible hydrolysis and existence of a slow rearrangement step after deformylation are, at first glance, surprising. However, previous studies with model peptides have shown that PDF can transfer the formyl group from one peptide to another^31^, indicating that a deformylated peptide can indeed act as a substrate for PDF. Deformylation as such does not trigger immediate release of the nascent chain from PDF, probably due to the similarity between the formylated and deformylated nascent chain. This is consistent with structural work indicating that most of the interactions between the peptide and PDF involve the peptide backbone and the methionine side chain, all of which remain unchanged during deformylation^31^. In addition, the presence of the formyl group is not monitored stringently by the enzyme, as substrates that are acetylated or carry a different N-terminal amino acid display a K_M_ value similar to fMet-peptides^25^. Also, the reversibility of the deformylation reaction agrees with studies on model peptides showing that adding increasing amounts of deformylated peptide into the reaction decreased the initial velocity^34^. Thus, the key features of the reaction mechanism are conserved between the model substrates and RNCs. The reason for rapid turnover with model substrates is that their affinity for PDF is low, promoting the dissociation of the complex, whereas with RNCs, PDF contacts with the ribosome favor retention of the nascent-chain at the enzyme active site, resulting in a k_cat_ that is 50-fold lower compared to model peptides. The slow rearrangement likely involves release of the N-terminus from the active site of PDF, since this step is followed by rapid dissociation of PDF from the ribosome. Different lengths of the nascent-chain as well as amino acid composition and secondary structure elements near the N-terminus can moderately influence the velocity of deformylation^2,20^. This is consistent with our analysis indicating that different rates of deformylation among RNC substrates depend on the rate-limiting conformational rearrangement of the nascent-chain which is reflected by the individual rate constants k_3_ for each RNC substrate in the kinetic model.

Our co-translational deformylation experiments show that the N-terminus of a nascent protein can be deformylated as soon as it emerges from the tunnel exit of a translating ribosome, consistent with previous studies^10,35^. This finding becomes important in the context of co-translational folding, in particular for small N-terminal domains that can fold within the ribosomal exit tunnel^36,37^. For these proteins, it is important that PDF acts early, before the N-terminus is buried within a folded domain^2,38^. Rapid deformylation followed by a slow rearrangement step may ensure that PDF holds the N-terminus of the nascent-chain for handover to downstream enzymes in nascent-chain processing. Specifically, the reversible deformylation and subsequent slow conformational rearrangement are responsible for the delay in methionine removal by MAP, which was observed in a coupled PDF-MAP assay^20^. Rapid PDF dissociation after the conformational rearrangement allows it to effectively vacate the primary binding site of MAP on the ribosome^16,18^, and subsequent binding of MAP is fast^20^, indicating that pre-organization of MAP or ‘priming’ at a secondary binding site on the ribosome is not required for efficient exchange of factors. Retention of the N-terminus by PDF may also prevent folding (or misfolding) of the nascent protein prior to recruitment of the ribosome-associated chaperone TF, which binds preferentially to longer nascent-chains, about 100 amino acids in length^39^. Therefore, PDF might help to bridge the gap between emergence of the nascent-chain from the ribosome and chaperone binding. A slow rearrangement that follows deformylation may introduce a relatively long pause into any number of events in the life of a nascent protein, including folding.

## Materials and methods

### PDF purification and labeling

PDF was expressed in BL21(DE3)pLysS (New England Biolabs) with a C-terminal His_6_-tag from a pET24-derived vector^19^. The enzyme was purified by Cobalt-Talon affinity chromatography followed by Q-Sepharose ion-exchange chromatography^2,40^. Purified PDF was stored in buffer A (25 mM HEPES, pH 7.5, 70 mM NH_4_Cl, 30 mM KCl, 7 mM MgCl_2_, 0.2 mM CoCl_2_, 1 mM TCEP, and 10% (v/v) glycerol) at − 80 °C. To prepare fluorescence-labeled PDF, a fusion protein comprised of PDF, a five amino-acid linker (LEGSY) and a C-terminal Mxe GyrA intein containing a chitin binding domain was overexpressed from a pTXB1 plasmid in BL21(DE3)pLysS (New England Biolabs). The fusion protein was affinity-purified on chitin resin in buffer B (20 mM Hepes at pH 7.4, 250 mM NaCl, 5 mM CoCl_2_) (according to Impact Kit, New England Biolabs) and intein cleavage (in buffer B without CoCl_2_) was induced by 2-mercaptoethanesulfonic acid (200 mM) overnight. After eluting protein from the column in buffer C (5 mM Bis–Tris at pH 6.5, 250 mM NaCl), a Bodipy-FL (Bpy) labeled peptide (CSDSK-Bpy, 2 mM) was added in 25-fold excess. The C-terminally labeled PDF(Bpy) was then purified by gel filtration using a Superdex75 column in buffer D (25 mM HEPES, pH 7.5, 70 mM NH_4_Cl, 30 mM KCl, 7 mM MgCl_2_, and 10% (v/v) glycerol) and stored in buffer D with 0.2 mM CoCl_2_ at − 80 °C.

### RNC preparation

RNCs were prepared using a reconstituted in-vitro translation system from *E. coli* as described^33^. Ribosomes from *E. coli* MRE600; initiation factors IF1, IF2 and IF3; elongation factors EF-Tu, EF-Ts and EF-G; and initiator fMet-tRNA^fMet^ were prepared according to standard protocols^41^. Total *E. coli* tRNA (Roche), was aminoacylated with a mixture of 19 amino acids plus [^14^C]Leu^36^. mRNAs were prepared by in-vitro transcription and purified by anion exchange chromatography^42^. Initiation complexes were prepared from 70S ribosomes, initiation factors, f[^35^S]Met-tRNA^fMet^ or f[^3^H]Met-tRNA^fMet^, and mRNA at 37 °C for 1 h, followed by translation in the presence of elongation factors and total aminoacyl-tRNA at 37 °C for 1 h. After completion of translation, RNCs were purified by sucrose gradient centrifugation^33^ and stored in buffer A. RNC concentrations were determined by radioactive counting of [^35^S] and/or incorporated [^14^C]Leu in the nascent-chain.

### Colorimetric deformylation assay

Deformylation of the model peptide fMLpNA (Bachem, 25–1500 μM) by PDF (0.01 μM) was monitored in buffer A at 37 °C^23^. For detection, *Aeromonas* aminopeptidase (0.8 U/ml) was included to digest the deformylated MLpNA and formation of pNA product was detected by 405 nm absorbance in a UV/Vis spectrometer (Lambda Bio+, Perkin Elmer) at 5 s time intervals.

### RNC deformylation assay

RNC deformylation was performed as described^2^. In brief, purified ^35^S-labeled RNCs were mixed with PDF in buffer A and incubated at 37 °C. The reaction was quenched by rapid boiling followed by digestion with proteinase K (Sigma-Aldrich, 20 mg/ml) at 37 °C overnight. The resulting amino acids were separated by thin-layer chromatography (TLC silica gel 60, Merck) with 3:1:1 butanol:acetic acid:water mobile phase, and a phosphorous imaging screen (imaging plate BAS-IP MS 2040, Fujifilm) was used for detection (Typhoon FLA 7000, GE Healthcare). The amounts of formylated and deformylated species were quantified by densitometry and the fraction of deformylated product was calculated. Oxidized Met and fMet, which were present in small amounts, were not included in the quantification. As reported earlier^2^, formylated RNC substrates carrying a nascent-chain with lysine in the second position were less sensitive to proteinase K digestion, resulting in formylated dipeptides that were included in the quantification. The samples of multiple-turnover time courses for DnaK75-RNC were quenched with 33% formic acid as described below for single-turnover experiments; the two quenching methods gave identical results.

To determine the multiple-turnover kinetics of deformylation, an excess of RNC was deformylated by catalytic amounts of PDF (Fig. 1a). The initial velocity, *V*_0_, was determined by linear fitting of the initial time points of deformylation time courses. After normalizing the initial velocity to the PDF concentration [PDF] and plotting it against the RNC concentration [RNC] (Fig. 1b), the Michaelis–Menten equation was used for fitting. For testing the reversibility of the reaction, TolB75-RNC (50 nM) was reacted with PDF (1 µM) for 10 min. Then, sodium formate (0.5–2 M) was added to the mixture and the reaction continued for 30 min. Workup and analysis were performed as described above. As controls, additions of buffer, sodium acetate (1 M), actinonin (1 mM) and actinonin (1 mM) together with sodium formate (up to 2 M) were tested.

### Single-turnover kinetics

Pre-steady-state kinetics was measured using a quench-flow apparatus (Kintek) at either 37 °C or 10 °C. ^35^S-labeled RNCs (10 nM) were reacted with saturating concentrations of PDF (25 μM) in buffer A and quenched with 33% formic acid. Samples were dried under vacuum at 45 °C, the remaining pellets were washed by resuspension in cold 75% acetone (− 20 °C) and centrifuged at 13.2×*g* for 15 min at 4 °C. After removing the supernatant, pellets were dried under vacuum at 45 °C and dissolved in buffer E (25 mM HEPES, pH 7.5, 70 mM NH_4_CL, 30 mM KCl, 7 mM MgCl_2_, 10 mM CaCl_2_, 2 mM TCEP) with 33 mg/ml proteinase K. Digestion was carried out overnight and Met and fMet were separated on TLC and quantified as above.

Rates measured under single-turnover conditions are, in principle, dependent on a number of elemental rate constants in Fig. 5. At these PDF concentrations, binding is not rate-limiting and rate constants k_1_ and k_−1_ do not contribute. Because the single-turnover reaction is faster than the multiple-turnover reaction by three orders of magnitude (k_app_ = 50 s^−1^ vs. k_cat_ = 0.05 s^−1^), steps after hydrolysis are too slow to contribute to the observed rate constant, k_app_. Based on this simplifying assumption, k_app_ for single-turnover deformylation then reduces to Eq. (1):

1 $${\mathrm{k}}_{\mathrm{app}}={\mathrm{k}}_{2}+ {\mathrm{k}}_{-2}$$

The end level of the reaction observed in the quench-flow experiments is obtained after the second step has reached equilibrium, but prior to any contribution by step three. This allows elemental rate constants k_2_ and k_−2_ to be calculated from Eq. (2):

2 $$\frac{\left[{\text{P}}\right]}{{\left[{\text{S}}\right]}_{0}}=\frac{{\mathrm{k}}_{2}}{{\mathrm{k}}_{2}+{\mathrm{k}}_{-2}}$$

where [P] and [S]_0_ are the product concentration and initial substrate concentration, respectively.

### PDF binding assay

Labeled PDF(Bpy) was mixed with ^3^H-labeled TolB75-RNC or 70S ribosomes in buffer A in a stopped-flow apparatus (SX-20MV, Applied Photophysics) at 10 °C. Fluorescence was monitored using an excitation wavelength of 470 nm (SX LED 470, Applied Photophysics) and a long-pass emission filter (Schott KV550). To improve the signal-to noise ratio, six to nine traces were averaged for each experiment. For dissociation experiments, RNCs or ribosomes were mixed with PDF(Bpy) and incubated for 2 min to allow complex formation and deformylation prior to the experiment. Dissociation was then monitored in the stopped-flow apparatus upon rapid mixing with an equal volume of PDF solution (15 μM, after mixing) or buffer; final concentrations of PDF(Bpy) and ribosomes/RNCs after mixing were 300 nM and 420 nM, respectively.

To determine the dissociation constant for PDF and ribosomes, PDF(Bpy) was mixed with increasing amounts of ribosomes and the fluorescence anisotropy was measured (FluoroMax-4, Horiba). The data were corrected for light scattering by ribosomes (ribosome-only control) and fitted to a hyperbolic function. To determine the rate constants k_1_ and k_−1_, the time courses of binding and dissociation at different ribosome concentrations were fitted globally using KinTek Explorer software^28,29^. The data set used for fitting was comprised of the PDF-70S binding titration and controls reflecting the extent of light scattering by ribosomes at various concentrations. The fitted rate constants describing the interaction of PDF with 70S ribosomes or RNCs at 10 °C were extrapolated to 37 °C as described (Eq. 3^30^).

3 $$\frac{{k}_{i}(37 \; ^\circ \mathrm{C})}{{k}_{\mathrm{TolB}75}(37 \; ^\circ \mathrm{C})}={\left(\frac{{k}_{i}(10\; ^\circ \mathrm{C})}{{k}_{\mathrm{TolB}75}(10 \; ^\circ \mathrm{C})}\right)}^{\frac{283.15\mathrm{ K}}{310.15\mathrm{ K}}}$$

The rates of single-turnover deformylation of TolB75-RNC at 37 °C and 10 °C, k_TolB75_ (37 °C) and k_TolB75_ (10 °C) were used as references to scale other rate constants (Table 2).

### Global fitting

Global fitting of multiple- and single-turnover experiments of TolB75-, DnaK75-, RNaseH75- and proOmpA75-RNCs was performed using KinTek Explorer software (Fig. 6) to the minimal model of deformylation (Fig. 5). Rate constants k_1_, k_−1_, k_2_, k_−2_, k_4_, k_−4_ were fixed to values determined by individual experiments. k_5_ and k_−5_ were the same for all substrates; k_5_ was fixed to a rate of 200 s^−1^. Even though formate is added to the reaction to keep the pH constant, the active species is expected to be formic acid. The concentration of active compound was fitted during global analysis to be 0.4 µM and 2.2 µM. Standard errors were determined by linear regression following the simulation of the rate constants (Table 3).

### Co-translational deformylation assay

A reconstituted in-vitro translation system was used to study cotranslational deformylation. Initiation complexes were formed with 70S ribosomes, mRNA as indicated and f[^35^S]Met-tRNA^fMet^ as described^42^. To start the reaction, purified initiation complexes (50 nM) were mixed with the translation machinery and PDF (2 μM) in buffer D (20 mM Tris, pH 7.5, 70 mM NH_4_Cl, 30 mM KCl, 3.5 mM MgCl_2_, 8 mM putrescine and 0.5 mM spermidine). Translation was carried out in the presence of EF-Tu (15 μM), EF-G (1 μM), EF-Ts (100 nM), 100 μM total aa-tRNA containing [^14^C]Leu-tRNA^Leu^, GTP (1 mM), phosphoenolpyruvate (3 mM) and pyruvate kinase (10 μg/ml). The reaction was quenched by rapid heating, and the extent of deformylation was determined as described above. In parallel, the progress of translation was monitored by analyzing translation products by Tris–glycine SDS-PAGE as described previously^42^. The translation reaction was stopped with 2% ammonia and the peptide was released from the RNC by incubating at 37 °C. In addition to quantification by radioactivity, translation was carried out with Bodipy-FL-labeled Met-tRNA^fMet^ at the N-terminal position which was detected by fluorescence imaging (Typhoon FLA 7000, GE Healthcare). The cellular concentration of PDF was calculated from the number of PDF molecules per cell (1300) and a cell volume of 10^–15^ l (Ref.^24^).

Time courses of co-translational deformylation (Fig. 7) were fitted by exponential functions taking into account a 40 s delay prior to the slow phase using the method reported previously^42^. Delays of 35 s or 45 s yielded the same rates, indicating the robustness of the fitted values. Apparent rates were compared using a one-way ANOVA (GraphPad Prism), which indicated a significantly lower rate for TolB50 than TolB75 and TolB100 (p = 0.013 and 0.0001, respectively), but no significant difference for TolB75 and TolB100 (p = 0.20).

## Supplementary Information

Supplementary Figures.
