# Supplementary material for: Kinetic control of nascent protein biogenesis by peptide deformylase
Source: Sci Rep. 2021 Dec 27;11:24457. doi: 10.1038/s41598-021-03969-3 (PMC8712518; doi:10.1038/s41598-021-03969-3)
Supplement: Supplementary file 1 — Supplementary Figures. [file 41598_2021_3969_MOESM1_ESM.pdf]

Supplementary Information for

Kinetic control of nascent protein biogenesis by peptide deformylase.

Lena A. K. Bögeholz, Evan Mercier, Wolfgang Wintermeyer and Marina V. Rodnina

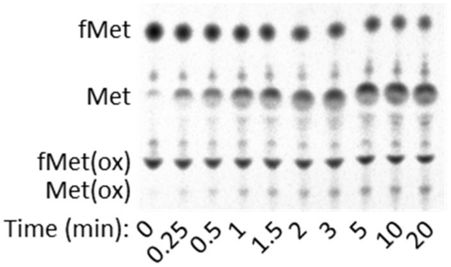

Figure S1. Monitoring RNC deformylation. Deformylation time course as monitored by TLC; exemplarily shown for RNaseH75-RNC. RNC (100 nM) was incubated with PDF (10 nM) at 37°C. Aliquots were quenched and fMet and Met were separated by TLC following protease digestion, and quantified by densitometry (Methods).

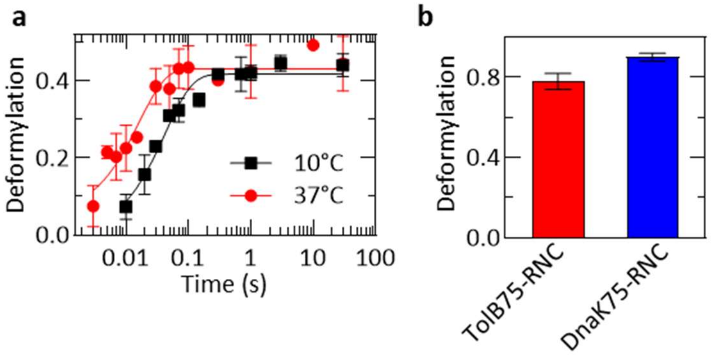

Figure S2. Single-turnover deformylation.

(**a**) Temperature dependence of deformylation kinetics. TolB75-RNC (10 nM) was mixed with PDF (25 µM) at 10°C. Single-exponential fitting yields k_{TolB75} (10°C) = (23 ± 3) s^{-1}. The time course measured at 37°C is shown for comparison; k_{TolB75} (37°C) = (60 ± 20) s^{-1} (Figure 2). Error margins represent the standard deviation of three independent experiments (n=3).

(**b**) End level of deformylation of TolB75-RNC and DnaK75-RNC (10 nM) after incubation with PDF (25 µM) for 10 min at 37°C.

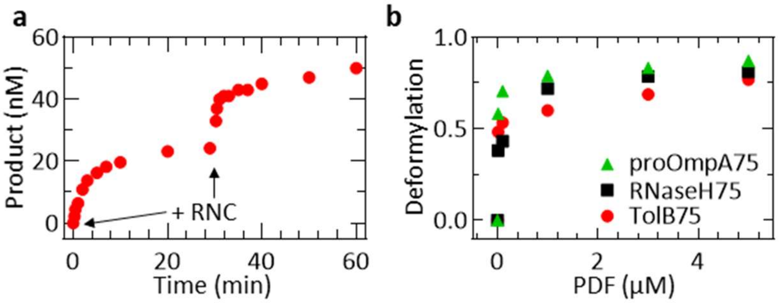

Figure S3. Activity of PDF and RNC.

(**a**) PDF activity. Substrate (50 nM TolB75-RNC) was added to PDF (10 nM) at the start of the reaction, and again after 30 min (arrows).

(**b**) RNC reactivity. End levels of deformylation time courses for proOmpA75-RNC, RNaseH75-RNC and TolB75-RNC (50 nM each) after 30 min at different PDF concentrations.

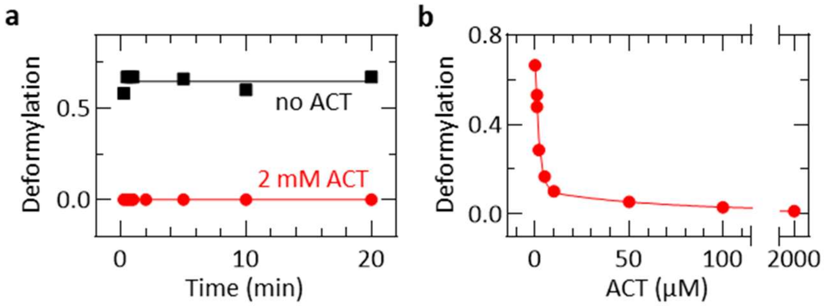

Figure S4: Inhibition by actinonin (ACT).

(**a**) TolB75-RNC (50 nM) was reacted with PDF (1 µM) with and without ACT.

(**b**) Increasing concentrations of ACT were added to a reaction of TolB75-RNC (50 nM) and PDF (1 µM). The reaction was quenched after 1 min.

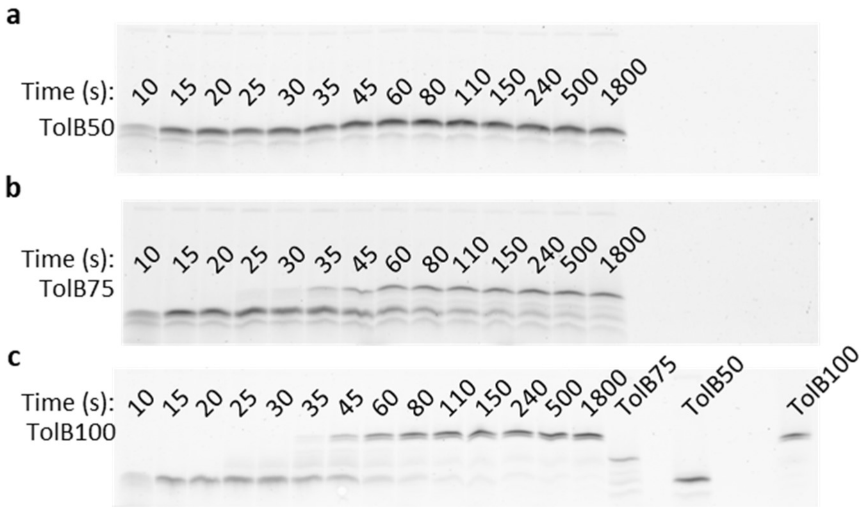

Figure S5: Translation of TolB50 (**a**), TolB75 (**b**) and TolB100 (**c**).

Initiation complexes (50 nM) were mixed with translation components and the reactions were quenched at indicated times. Translation products were separated by Tris-Tricine SDS-PAGE. In (**c**) final translation products of TolB75, TolB50 and TolB100 were loaded for comparison.

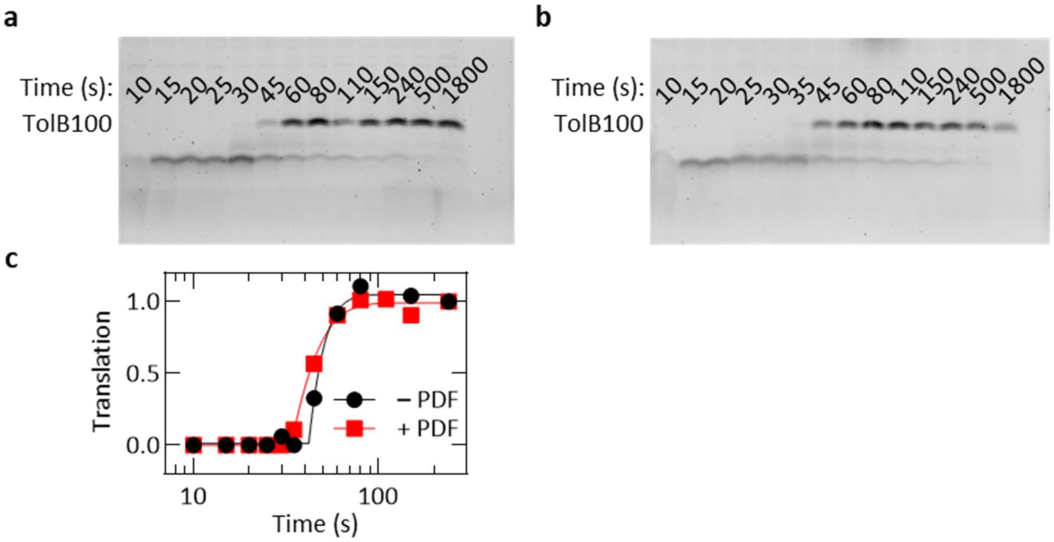

Figure S6: Translation in the presence of PDF.

(**a**) Translation time course of TolB100. TolB100 initiation complex (25 nM) was mixed with components of the translation system to start translation. Samples were quenched at the indicated time points by 2% ammonia and resolved by electrophoresis.

(**b**) Translation of TolB100 in the presence of PDF (2 µM).

(**c**) Quantification of translation gels in (**a**) and (**b**). Time points were standardized by lane intensities and normalized to the 240 s time point.
